# A Novel Image-Encryption Scheme Based on a Non-Linear Cross-Coupled Hyperchaotic System with the Dynamic Correlation of Plaintext Pixels

**DOI:** 10.3390/e22070779

**Published:** 2020-07-17

**Authors:** Wenjin Hou, Shouliang Li, Jiapeng He, Yide Ma

**Affiliations:** School of Information Science and Engineering, Lanzhou University, Lanzhou 730000, China; houwj17@lzu.edu.cn (W.H.); hejp17@lzu.edu.cn (J.H.)

**Keywords:** cross-coupled hyperchaotic map, digital image encryption, dynamic correlation

## Abstract

Based on a logistic map and Feigenbaum map, we proposed a logistic Feigenbaum non-linear cross-coupled hyperchaotic map (LF-NCHM) model. Experimental verification showed that the system is a hyperchaotic system. Compared with the existing cross-coupled mapping, LF-NCHM demonstrated a wider hyperchaotic range, better ergodicity and richer dynamic behavior. A hyperchaotic sequence with the same number of image pixels was generated by LF-NCHM, and a novel image-encryption algorithm with permutation that is dynamically related to plaintext pixels was proposed. In the scrambling stage, the position of the first scrambled pixel was related to the sum of the plaintext pixel values, and the positions of the remaining scrambled pixels were related to the pixel values after the previous scrambling. The scrambling operation also had a certain diffusion effect. In the diffusion phase, using the same chaotic sequence as in the scrambling stage increased the usage rate of the hyperchaotic sequence and improved the calculation efficiency of the algorithm. A large number of experimental simulations and cryptanalyses were performed, and the results proved that the algorithm had outstanding security and extremely high encryption efficiency. In addition, LF-NCHM could effectively resist statistical analysis attacks, differential attacks and chosen-plaintext attacks.

## 1. Introduction

With the rapid development of network communication, the internet of things and artificial intelligence, information exchange through text, audio and video is becoming more and more frequent, which brings great convenience and shortcuts to people’s life and work. At the same time, as the interactions of this information is carried out on public channels, how to ensure the security of information is related to the vital interests of individuals, teams and even the country. As an aspect of multimedia information, image information has been given more and more attention in network communication. As mentioned above, traditional encryption methods, such as data encryption standard (DES) and advance encryption standard (AES) algorithms, are not suitable because image information has different special properties compared with text information, such as a large amount of data, a strong correlation between data and heavy data redundancy. The ergodicity and quasi-randomness of chaotic systems are very suitable for the encryption field.

Matthews [1] first applied a chaotic system to text encryption. With Fridrich [2] first applying a chaotic system to digital image encryption in 1998, and putting forward the scrambling diffusion structure, a large number of chaotic image-encryption algorithms based on this structure were proposed. Some algorithms are based on pixel level scrambling and permutation [3,4], some are based on bit level scrambling [5,6,7,8], and some are based on the improvement of the diffusion algorithm [9,10,11]. Certain encryption algorithms add plaintext correlation [12,13,14] to resist known-plaintext attacks.

In image encryption based on chaos, a chaos system is generally used to generate a random-like chaotic sequence as the key stream of scrambling and diffusion in the encryption algorithm. Therefore, the selection of the chaos system is particularly important. As the characteristics of low dimension continuous chaotic systems, such as few system parameters, narrow chaotic intervals and many periodic windows, many references [15,16,17] constructed high dimension continuous chaotic systems based on a low dimension chaotic system, and then applied this to image encryption to obtain a better encryption effect. However, although the dynamic characteristics of the high-dimensional continuous chaotic system were more abundant, the system parameters were also more. At the same time, the calculation complexity is increased when outputting the chaotic sequence, which increases the calculation complexity and the time cost of the encryption algorithm.

Therefore, many researchers pay attention to discrete chaotic systems. In the same way, because the one-dimensional discrete chaotic system [18,19,20] has insufficient dynamic characteristics, a too narrow chaotic interval and a small periodic window, it was confirmed in [21,22] that the one-dimensional discrete chaotic system is vulnerable to enemy attack, which makes the encryption algorithm vulnerable. Therefore, researchers designed a new discrete chaotic system [18,19,23]. By increasing the dimension and structure complexity of the system, the complexity of the system is increased, and the quasi-randomness of the chaotic sequence generated by the system is enhanced. Literature [24] proposed a block encryption algorithm based on continuous three-dimensional chaotic system. Compared with discrete hyperchaotic systems, this system produces chaotic sequences of the same length with less randomness.

At present, many encryption algorithms based on chaos were proved to be unsafe and easy to be cracked by cryptanalysts [21,25,26,27,28,29,30,31]. The reason is that most of these encryption algorithms are not sensitive to plaintext changes and have weak key design. The most important factor is that the key stream used in the scrambling and diffusion process is completely dependent on the key. That is to say, although these algorithms are related to plaintext when they form a key, as long as the key does not change, the key stream generated by the key will not change in the scrambling and diffusion stage, so when the same key is used to encrypt different plaintext images, the key stream is not secure. An attacker can choose to bypass the direct attack key, through a known-plaintext attack [26,27,28,29] and select a plaintext attack [26,27,29,30,31] to obtain the key stream, to achieve cracking. Therefore, to increase the security of the encryption algorithm, the key flow should be related to plaintext as much as possible.

In [12], the parameters and initial values of the other three chaotic maps were generated by iterating the logistic map M times, in which M was related to the first pixel value of the plaintext image and was related to the plaintext image. Generally, an image-encryption algorithm based on chaos uses multiple chaotic state sequences generated by a chaotic system, some of which are used to scramble the sequence of pixel bits, pixels or image blocks, and then uses other chaotic state sequences to confuse the corresponding bits or pixel values in the scrambling stage. One of the disadvantages of these algorithms is that the generation of chaotic state sequences consumes a large amount of computing resources and does not make full use of these sequences.

Therefore, Chen et al. [32] proposed a novel chaotic image-encryption algorithm with a dynamic state variable selection mechanism. The algorithm uses the same set of chaotic state sequences in the scrambling and diffusion stage, which overcomes the shortcomings of the algorithm in resisting known-plaintext attacks and selected plaintext attacks. However, in this document, three chaotic state sequences must be generated to scramble by correlating with plaintext pixels. In fact, the length of each chaotic state sequence to be used is related to the plaintext image, and the length is uncertain. Therefore, when generating the chaotic sequence, it must be more than the actual number of pixels, which will cause a waste of state resources. Although the scrambling operation has a diffusion effect, if the attacker attacks from the last plaintext pixel, the encryption algorithm loses its diffusion effect. The encryption algorithm proposed in [24] has nothing to do with the plaintext in the key generation and scrambling process. It cannot resist known-plaintext attacks and selective plaintext attacks, so the security is not high.

Based on the above description, the current image-encryption algorithm based on chaos has the following disadvantages: (1) The dynamic characteristics of the chaos system used are not complex enough, the chaotic parameter range is narrow and there is a periodic window, resulting in a narrow key space. (2) The chaotic state sequence is used as the key stream in the scrambling and diffusion stage, which only depends on the key, the key stream cannot resist the known information In the scrambling and diffusion stage, and different mixed state sequences are typically used, which makes the use rate low and increases the computational complexity of the algorithm.

In view of the above shortcomings, we proposed an image-encryption scheme based on a hyperchaotic system with the dynamic correlation of plaintext pixels. This system has the following advantages: (1) Based on the cross-coupled chaotic map proposed in [33], a non-linear cross-coupled hyperchaotic map is designed, which has a wider hyperchaotic interval, no periodic window and more complex dynamics. (2) In the scrambling stage of the encryption algorithm, only one chaotic state sequence with the same number of plaintext pixels is used to realize the position transformation of the chaotic state sequence related to the plaintext pixels, which increases its resistance to known-plaintext attacks and selective plaintext attacks. (3) In the diffusion stage of the encryption algorithm, the scrambling order is used. The same chaotic sequence increases the use of the chaotic state mapping and reduces the complexity of the algorithm.

The rest of this paper is organized as follows: In the second part, the logistic Feigenbaum non-linear cross-coupled hyperchaotic map (LF-NCHM) model is proposed, and its chaotic characteristics are analyzed and compared with the existing chaotic model by using a trajectory diagram, bifurcation diagram, Lyapunov index and permutation entropy. In the third part, we propose an image-encryption algorithm, based on the LF-NCHM model, which is dynamically related to plaintext pixels. In the fourth part, the security and time complexity of the algorithm are analyzed. Finally, the research results are summarized.

## 2. Non-Linear Cross-Coupled Chaotic Map

**Figure 1:**
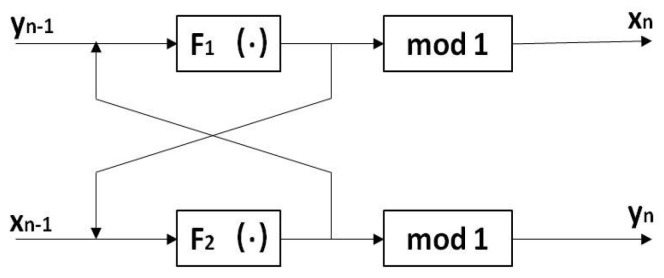
Cross-coupled chaos model structure diagram.

In 2014, Paral et al. [33] proposed a cross-coupled chaotic map, whose structure is shown in Figure 1.

As can be seen from Figure 1, the model has two inputs and two outputs. Given the initial value of $\left(x_{0},y_{0}\right)$, the output of $F_{1}$ (·) is the input of $F_{2}$ (·), and the input of $F_{2}$ (·) is the input of $F_{1}$ (·). If the output variable is greater than zero, the result is the decimal part of the output variable; if the output variable is less than zero, the result is the sum of the absolute value and x of the largest integer less than the output variable. The mathematical expression of the model is shown in Formula (1).

(1) $$\left\{\begin{array}{c} x_{n}=mod\left(F_{1}\left(y_{n-1}\right),1\right)\\ y_{n}=mod\left(F_{2}\left(x_{n-1}\right),1\right) \end{array}\right.$$

where $F_{1}$ (·) and $F_{2}$ (·) are the given chaotic map, mod(x,1) is the modulo of *x* to 1. $F_{1}$ (·), $F_{2}$ (·) can be chosen as any one-dimensional chaotic map, and its combination form can be changed when the output is fed back to the input in the iterative process, so as to obtain a better chaotic map. Therefore, the model has strong expansibility.

**Figure 2:**
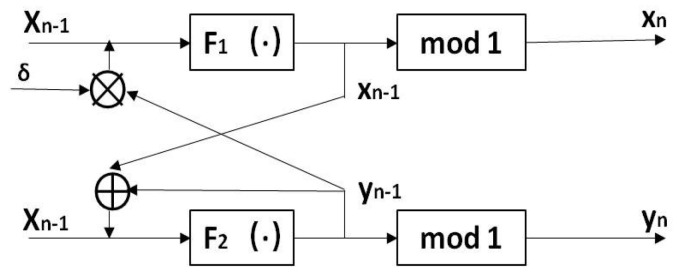
Structural diagram of a non-linear cross-coupled hyperchaotic map (NCHM).

To obtain a more dynamic chaotic model, the cross-coupled chaotic model was improved. In the iterative feedback, a non-linear cross-coupled hyperchaotic map (NCHM) model was designed, as shown in Figure 2.

In Figure 2, the cross-coupled chaotic model is improved at the feedback input, where the + sign represents the addition of two input terms, the × sign represents the multiplication of two input terms, and the $F_{1}$ (·), $F_{2}$ (·) can be selected as any one-dimensional chaotic map. When $F_{1}$ (·) is selected as the logistic map and $F_{2}$ (·) is selected as the Feigenbaum map, the LF-NCHM is formed, and the mathematical expression is shown in (2).

(2) $$\left\{\begin{array}{c} x_{n}=mod\left(\mu {*\left(\delta *y\right.}_{n-1}\right)\left.*\left(1-{\delta *y}_{n-1}\right),1\right)\\ y_{n}=mod\left(\lambda *sin\left(\pi *\left(x_{n-1}+y_{n-1}\right)\right),1\right) \end{array}\right.$$

where the parameter μ∈(0,4]. After many experiments, we found that the model demonstrated good performance when λ=5, so for the rest of this paper λ is set to 5.

### 2.1. Performance Evaluation of LF-NCHM Model

Chaos is a complex non-linear system, and the methods of studying chaos are both qualitative and quantitative. Without a loss of generality, to evaluate the performance of LF-NCHM, we analyzed the chaos map using the trajectory, bifurcation diagram, Lyapunov exponent and permutation entropy.

#### 2.1.1. Trajectory

**Figure 3:**
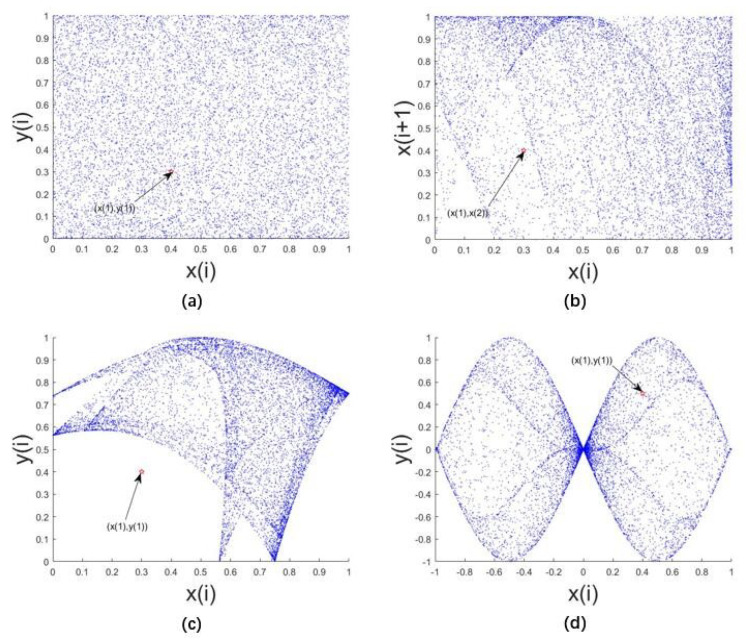
Trajectories of the (**a**) logistic Feigenbaum non-linear cross-coupled hyperchaotic map (LF-NCHM), (**b**) FL-NMLD, (**c**) 2D-SLMM and (**d**) 2D-SIMM.

In the limited phase space, the larger and more uniform the motion trajectory of the system, the better the random performance of the state variables of the system over time, and the better traversal of the limited phase space. Figure 3 shows the trajectory of the LF-NCHM, Nonlinearly Modulated Logistic Map with Delay(FL-NMLD) [34], 2D Sine Logistic modulation map(2D-SLMM) [19] and two-dimensional Sine ICMIC modulation map(2D-SIMM) [35]. The red dot in the figure represents the initial point of the system iteration. Through comparison, it can be seen that although the track distribution of FL-NMHMD is larger than that of 2D-SLMM and 2D-SIMM, covering almost the whole phase plane, FL-NCHM covers the entire x-axis and y-axis two-dimensional interval composed of [0,1], and the distribution of track points is very uniform. This means that the output of LF-NCHM has better ergodicity; therefore, a sequence with better random characteristics can be obtained through the system.

#### 2.1.2. Bifurcation Diagram

**Figure 4:**
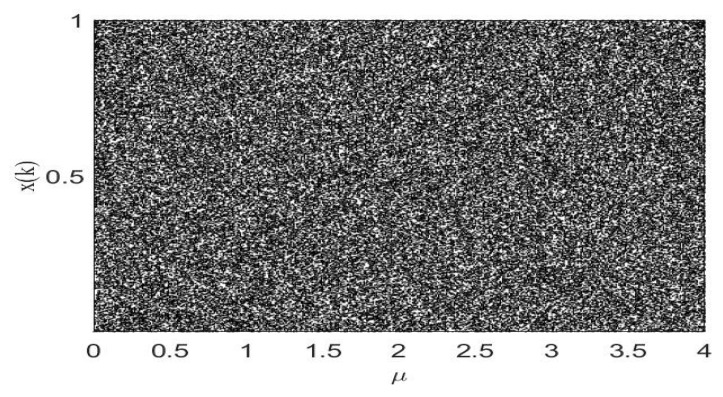
The bifurcation diagram of the LF-NCHM.

A bifurcation diagram indicates that when the control parameters of the system change, the motion state of the system will change essentially, and the system state will change discontinuously in the parameter range. The bifurcation diagram can directly observe the evolution of the system with parameters. Figure 4 shows the bifurcation diagram of LF-NCHM. It can be seen from the figure that in different parameter ranges, the output trajectory points of LF-NCHM are evenly distributed, occupying the [0,1] range, and the system is in a chaotic state.

#### 2.1.3. Lyapunov Exponent

The Lyapunov index is mainly used to describe the characteristics of the system movement. The positive and negative sum of its values along a certain direction can indicate the speed of the average divergence or convergence of the system along the direction of the adjacent orbits in the attractor for a period of time. When the system is in chaos, there must be a positive Lyapunov index. Therefore, the Lyapunov index can be used as the criterion for whether the system is chaotic and whether it is in a chaotic state under the current parameter setting. Hyperchaotic systems are defined as having two or more positive Lyapunov exponents. Through computer simulation, LF-NCHM has two positive Lyapunov exponents and the same dynamic behavior as the bifurcation diagram; thus, it is a hyperchaotic system.

**Figure 5:**
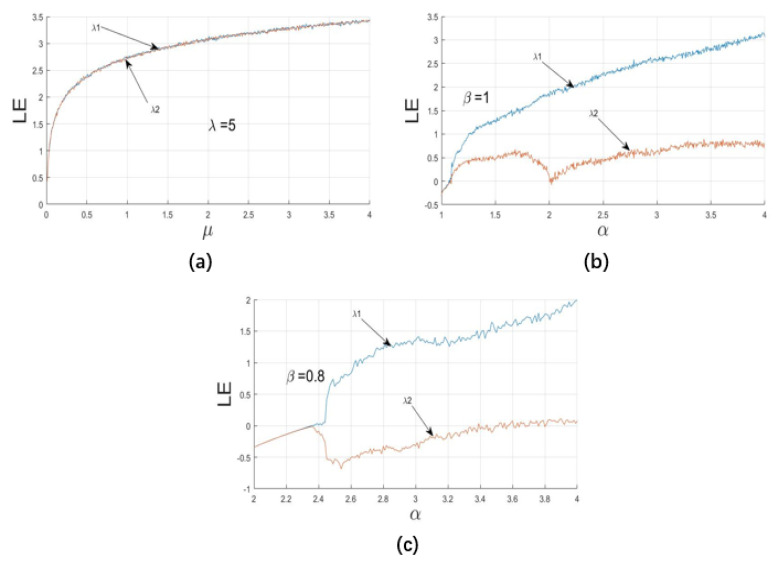
Lyapunov Exponent value of the (**a**) LF-NCHM, (**b**) FL-NMLD and (**c**) DLCL.

Figure 5a–c show the Lyapunov exponents of LF-NCHM, FL-NMLD and delay and linearly coupled Logistic chaotic map(DLCL). For LF-NCHM, μ∈(0,4], the parameter μ is increased by 0.01 each time, and the Lyapunov index is calculated. In the whole range of parameters, the system has two Lyapunov exponents λ${}_{1}$, λ${}_{2}$, which are greater than zero; therefore, the system is hyperchaotic. For FL-NMLD and DLCL, α∈(2,4), the parameter α is increased by 0.01 each time, and the Lyapunov index is calculated. From Figure 5b,c, these two maps have two Lyapunov exponents; however, on the whole range of parameters, the Lyapunov exponents have positive, negative and zero values—that is, there are hyperchaotic, chaotic and periodic intervals. Compared with FL-NMLD and DLCL, LF-NCHM has a wider hyperchaotic parameter range, in which there is no periodic window; in addition, LF-NCHM has a larger Lyapunov index value than FL-NMLD and DLCL, indicating that LF-NCHM is more sensitive to the initial value and initial conditions, that predicting the chaotic sequence is more difficult, and that LF-NCHM can generate a larger key space.

#### 2.1.4. Permutation Entropy

**Figure 6:**
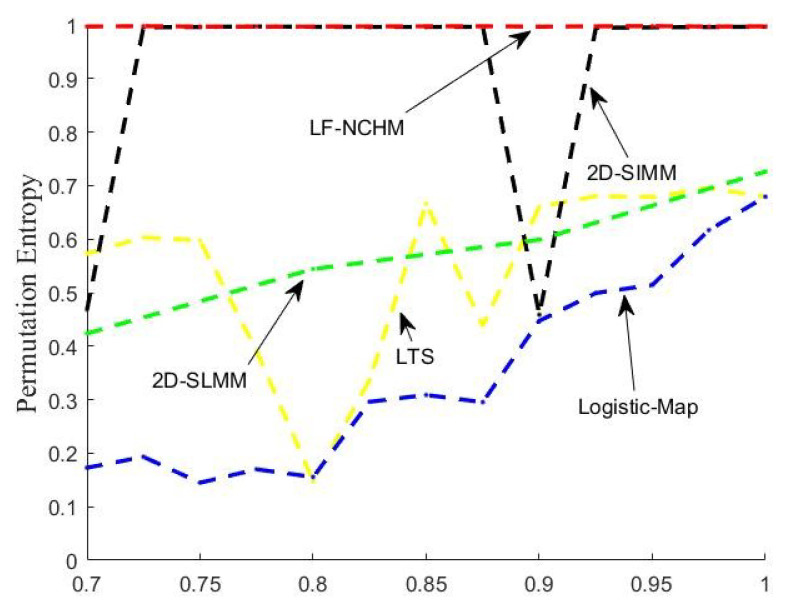
The permutation entropy (PE) of LF-NCHM, 2D-SIMM, 2D-SLMM, LTS and logistic map.

Permutation entropy (PE) [36] is an effective method to measure the complexity of the motion state of a non-linear system. The larger the value of permutation entropy is, the more difficult it is to predict the sequence generated by a chaotic system, which indicates that the dynamic behavior is more complex. In computer simulation, we used the method proposed in [36] to calculate the PE. Figure 6 shows the permutation entropy of LF-NCHM, 2D-SIMM, 2D-SLMM, LTS and logistic map parameters in the range of [0.7,1]. From the figure, the permutation entropy of LF-NCHM is close to the ideal value of 1, and there is no periodic window. 2D-SIMM also has better permutation entropy; however, there is a periodic window, and the value is not stable enough—other chaotic systems have smaller permutation entropy, and the value changes violently. In conclusion, LF-NCHM has better non-linear characteristics and more complex dynamic behavior.

## 3. The Image-Encryption Algorithm Related to Plaintext Pixels in the Scrambling Process

In the encryption algorithm, the scrambling and diffusion processes are conducted separately, and the scrambling process is cleverly designed. Except for the first pixel in the plaintext image, each pixel position is associated with the previous pixel value of the pixel; thereby, each pixel has the diffusion effect. In the scrambling stage, first, the chaotic sequence used for scrambling is generated, which is sorted in order of size to form a sequence of image pixels. Then, the image is transformed into a one-dimensional sequence with M*N elements. Finally, according to the value of the previous pixel, we determined whether the position sequence of the current pixel was selected from the left or the right of the sequence. Therefore, the previous pixel value can affect the position selection of subsequent pixels. Thus, there is a certain diffusion effect.

In the diffusion stage, including two rounds, to make the correlation between pixels more complex, the current pixel is generally related to the previous two directions. To improve the use of the chaotic sequence, the confusion sequence is the chaotic sequence produced in the scrambling stage. In the two-dimensional pixel plane of the image, we started from the upper left corner, spread from left to right, top to bottom, so that the current pixel was related to its left and upper edge. Then, we performed the second round, starting from the lower right corner, right to left, bottom to top, so that the current pixel was related to its right and lower edge.

**Figure 7:**
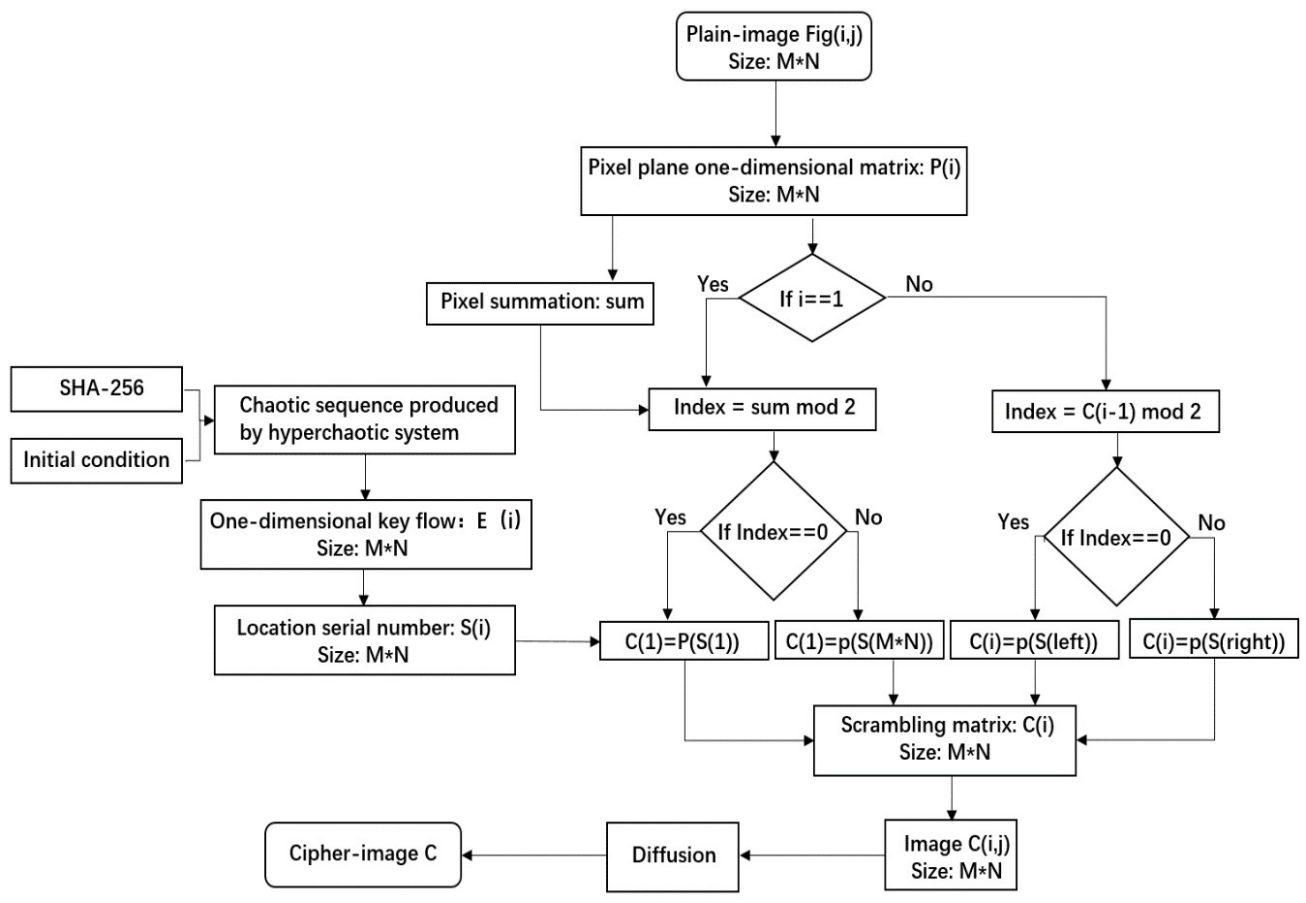
The flow diagram of the proposed algorithm.

The specific flow of the algorithm is shown in Figure 7.

In the scrambling phase, the pixel position is related to each pixel value of the plaintext image, and has a certain diffusion effect, so this enhances the ability of the algorithm to resist the attack of selecting plaintext.

### 3.1. The Generation of Keys and Hyperchaotic Sequences

In the process of generating the key, we used the hash algorithm SHA256 to make the key generation related to the plaintext image; thus, the generated key is a dynamic key, the length of the key *K* is 256 bits, and the key is highly related to the plaintext. Any pixel change of the plaintext will cause a key change. Based on the key K, the intermediate variables were designed to generate the initial value of LF-NCHM, so that the change of each initial value can affect other initial values; therefore, the initial value has a strong key sensitivity. After the initial conditions are generated, the chaotic sequences needed for the scrambling and diffusion processes can be obtained by introducing them into LF-NCHM. The specific steps are as follows:

1. Use the image file to generate hash values, Obtain the *K = k${}_{1}$, k${}_{2}$, ……, k${}_{32}$*. They are composed of 8 bits of data.
2. Calculate the intermediate variables *h${}_{1}$, h${}_{2}$, h${}_{3}$, h${}_{4}$* through *k${}_{1}$, k${}_{2}$, ……, k${}_{32}$*. The calculation rules are shown in Formula (3).
  (3) $$\left\{\begin{array}{c} h_{1}=\frac{\left(k_{25}⊕k_{26}⊕\ldots ⊕k_{32}\right)}{256}\\ h_{2}=h_{1}+\frac{\left(k_{1}⊕k_{2}⊕\ldots ⊕k_{8}\right)}{256}\\ h_{3}=h_{2}+\frac{\left(k_{17}⊕k_{18}⊕\ldots ⊕k_{24}\right)}{256}\\ h4=h_{3}+\frac{\left(k_{9}⊕k_{10}⊕\ldots ⊕k_{16}\right)}{256}. \end{array}\right.$$
3. Calculate the initial values μ, λ, *x${}_{0}$ and y${}_{0}$* according to *h${}_{1}$, h${}_{2}$, h${}_{3}$ and h${}_{4}$.* The calculation rules are shown in Formula (4).
  (4) $$\left\{\begin{array}{c} \mu =mod\left(\mu_{0}^{{}^{\prime }}+\frac{\left(h_{1}+h_{2}\right)}{256},4\right)\\ \lambda =mod\left(\lambda_{0}^{{}^{\prime }}+\frac{\left(h_{2}+h_{3}\right)}{256},6\right)\\ x_{0}=mod\left(x_{0}^{{}^{\prime }}+\frac{\left(h_{3}+h_{4}\right)*10^{14}}{256},1\right)\\ y_{0}=mod\left(y_{0}^{{}^{\prime }}+\frac{\left(h_{4}+h_{1}\right)*10^{14}}{256},1\right) \end{array}\right.$$
  where *μ${}_{0}^{{}^{\prime }}$, λ${}_{0}^{{}^{\prime }}$, x${}_{0}^{{}^{\prime }}$ and y${}_{0}^{{}^{\prime }}$* are the initial values given. In the algorithm of this chapter, μ${}_{0}^{{}^{\prime }}$ = 3.0, λ${}_{0}^{{}^{\prime }}$ = 4.5, *x${}_{0}^{{}^{\prime }}$* = 0.4 and *y${}_{0}^{{}^{\prime }}$* = 0.3.
4. Bring the initial conditions generated by the hash algorithm into LF-NCHM to produce a sequence of the required length.

### 3.2. A Scrambling Method Based on Pixel Values

Without losing generality, we used a typical chaos-based cryptosystem architecture. This structure consists of two stages: the scrambling stage and the diffusion stage.

Our proposed scrambling method is related to pixel values. All operations were carried out in a one-dimensional plane. The first scrambling pixel is related to the sum of the pixels, and the remaining pixels are related to the previous one. Through this operation, all pixels are related to each other. The specific steps are as follows:

1. Convert the plaintext image *Fig* with the size of M*N into the one-dimensional vector *P(i)*, and the size of the one-dimensional vector is M*N.
2. According to the dynamic secret key *K* related to the plaintext and hyperchaotic system LF-NCHM mentioned above, generate a one-dimensional key stream *E(i)* with the size of M*N.
3. Sort *E(i)*, and a one-dimensional vector of position sequence number with the size of M*N is obtained. The vector of the position sequence number is named *S(i)*. An example of this rule is shown in Figure 8.
  For example:

**Figure 8:**
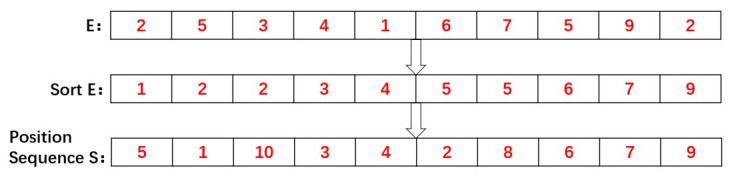
Example of the location sequence number generation.
4. Calculate the sum of the pixel values and index for the plaintext image. *index = sum mod 2*.
5. Use the MATLAB library function *zeros* to produce a one-dimensional vector *C(i)* of size M*N, which is used to save the pixel scrambling results.
6. Judge the *index*. If the *index* is equal to zero, select the location information from the left side of S(i), C(1)=P(S(1)). If the *index* is equal to one, choose to take the location information from the right side of S(i), C(1)=P(S(M*N)). Figure 9 shows this operation.
7. Calculate *index*. index=C(i−1) *mod* *2*, *i ≥ 2*. If the *index* is equal to zero, select the location information from the left side of S(i). C(i)=P(S(left)), and *left* represents the latest sequence number of location sequence from the left. If *index* is equal to one, select the location information from the right side of S(i). C(i)=P(S(right)), *right* represents the latest sequence number of the position sequence starting from the right side. Repeat the operation until the assignment of *C*(M*N) is completed.
8. Restore the one-dimensional vector C(i) to the two-dimensional matrix C(i,j) with the size of M*N, and the scrambling process is completed.

### 3.3. Diffusion Process

**Figure 10:**
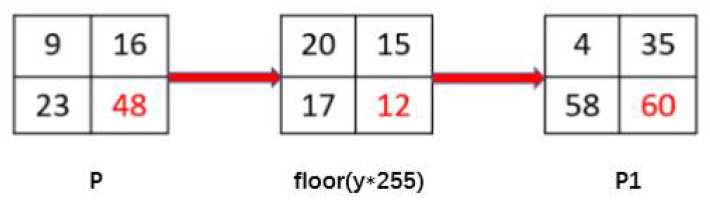
Example of the diffusion process.

The purpose of the diffusion operation is to change the value of the pixel, to reduce the correlation of the adjacent pixels. Diffusion includes pixel-by-pixel diffusion and block diffusion. Here, the algorithm adopted two rounds of pixel-by-pixel diffusion. In the two-dimensional pixel plane of the image, we started from the upper left corner, spread from left to right, top to bottom, so that the current pixel was related to its left and upper sides; then, we performed the second round, starting from the lower right corner, right to left, bottom to top, so that the current pixel was related to its right and lower sides. The steps are as follows:

1. According to the generation rules of the key and hyperchaotic sequences, generate sequences x(i),y(i) with the size of M*N. The sequences x(i),y(i) are transformed into two-dimensional matrixes x(i,j),y(i,j) of size M*N.
2. Diffuse the scrambled image C(i,j). First, from the lower right corner of the image, from right to left, from bottom to top. The current pixel is associated with the adjacent pixels on the right and bottom, and the intermediate image *P1* is obtained. The specific operations are as follows:
  fori=M:−1:1
  forj=N:−1:1
  ifi==M&&j==N
  P1(i,j)=C(i,j)⊕floor(y(i,j)∗255)
  elseifi==M
  P1(i,j)=C(i,j)⊕P1(i,j+1)⊕floor(y(i,j)∗255)
  elseifj==N
  P1(i,j)=C(i,j)⊕P1(i+1,j)⊕floor(y(i,j)∗255)
  else
  P1(i,j)=C(i,j)⊕P1(i,j+1)⊕P1(i+1,j)⊕floor(y(i,j)∗255) where ⊕ indicates that binary values corresponding to two numbers are exclusive or by bits, and floor(x) indicates the largest integer not greater than *x*. *P1* is the first round of diffusion of encrypted images.
  Here we use four pixels as an example to give the calculation method. Figure 10 shows the operation process.
  P1(2,2)=C(2,2)⊕floor(y(2,2)∗255)=60
  P1(2,1)=C(2,1)⊕P1(2,2)⊕floor(y(2,1)∗255)=58
  P1(1,2)=C(1,2)⊕P1(2,2)⊕floor(y(1,2)∗255)=35
  P1(1,1)=C(1,1)⊕P1(1,2)⊕P1(2,1)⊕floor(y(1,1)∗255)=4
3. Carry out the second round of diffusion on *P1*. Starting from the upper left corner of the image, from left to right, and from top to bottom, the current pixel is associated with the adjacent pixels on the left and upper sides to obtain the encrypted image *P2*. The specific operations are as follows:
  fori=1:1:M
  forj=1:1:N
  ifi==1&&j==1
  P2(i,j)=P1(i,j)⊕floor(x(i,j)∗255)
  elseifi==1
  P2(i,j)=P1(i,j)⊕P2(i,j−1)⊕floor(x(i,j)∗255)
  elseifj==1
  P2(i,j)=P1(i,j)⊕P2(i−1,j)⊕floor(x(i,j)∗255)
  else
  P2(i,j)=P1(i,j)⊕P2(i,j−1)⊕P2(i−1,j)⊕floor(x(i,j)∗255)
  This process is the reverse of Step 2.

### 3.4. Decryption Process

According to our encryption scheme, the operation to obtain the decrypted image is as follows:

**Input.** Security key *K =*{*μ${}_{01}$, λ${}_{01}$, x${}_{01}$, y${}_{01}$, μ${}_{02}$, λ${}_{02}$, x${}_{02}$, y${}_{02}$*} and encrypted image *P2*.

1. Using key *K*, the hyperchaotic sequences x(i),y(i) for inverse diffusion and the hyperchaotic sequence E(i) for the inverse scrambling are obtained.
2. Through x(i), the first round of reverse diffusion is carried out, and the result is *P3*. The method is as follows.
fori=M:−1:1
forj=N:−1:1
ifi==1&&j==1
P3(i,j)=P2(i,j)⊕floor(x(i,j)∗255)
elseifi==1
P3(i,j)=P2(i,j)⊕P2(i,j−1)⊕floor(x(i,j)∗255)
elseifj==1
P3(i,j)=P2(i,j)⊕P2(i−1,j)⊕floor(x(i,j)∗255)
else
P3(i,j)=P2(i,j)⊕P2(i,j−1)⊕P2(i−1,j)⊕floor(x(i,j)∗255)
  This process starts from *i = M and j = N*, ⊕ indicates that the binary values corresponding to the two numbers are bitwise exclusive or, floor(x) is the largest integer not greater than *x*, and *P3* is the transformed image.
3. Carry out the second round of reverse diffusion through y(i), and obtain *P4*. The method is similar to Step 2 and will not be repeated.
4. According to the scrambling method, *P4* is transformed into a one-dimensional matrix *C*${}^{{}^{\prime }}$ and the pixel sum of *P4* is calculated.
5. Sort E(i) to obtain the position serial number $S^{{}^{\prime }}\left(i\right)$.
6. Calculate the *index’*. $index{}^{\prime }=sum\;mod\;2,i=1$. If *index’* equals zero, $P(S{}^{\prime }(1))=C{}^{\prime }(1)$. Otherwise, *P*(*S’*(M*N))*= C’*(*1*).
7. Calculate the *index’*. $index{}^{\prime }=C^{\prime }\left(i-1\right)\;mod\;2,i>=2$. If *index’* equals zero, *P*(*S’*(*left*))*=C’*(*i*)*, left = left +*1. Otherwise, $P(S{}^{\prime }(right))=C{}^{\prime }(i),right=right-1$. Until *i* is equal to M*N.
8. Restore vector *P(i)* to a decrypted image *P* of size M*N.

## 4. Simulation Results and Attack Test

### 4.1. Simulation Results

**Figure 11:**
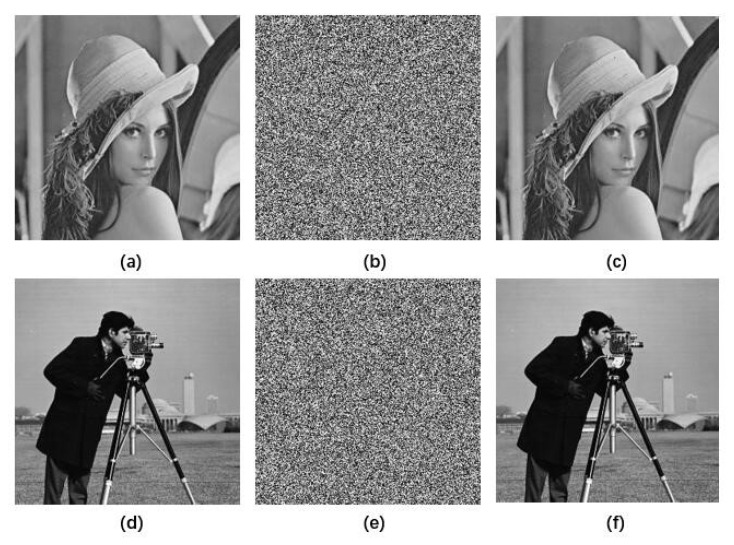
The simulation test results. (**a**–**c**) are the original image, encrypted image and decrypted image of the Lena image, respectively. (**d**–**f**) are the original image, encrypted image and decrypted image of the Cameraman image, respectively.

We used the Lena and Cameraman gray images with a size of 256 × 256 to test. The results are shown in Figure 11. (a–c) corresponding to the Lena original plaintext image, encrypted image and decrypted image; and (d–f) corresponding to the cameraman original plaintext image, encrypted image and decrypted image. The encrypted image is a kind of random image, and no effective information can be read out from the perspective of human vision, which demonstrates the effectiveness of the encryption. Then, the original plaintext image can be extracted from these encrypted images using the decryption algorithm. From the result of the decryption, the information of the original image can be undistorted without any loss and change, which shows the validity and feasibility of the decryption algorithm.

### 4.2. Statistical Analysis

#### 4.2.1. Histogram Analysis

**Figure 12:**
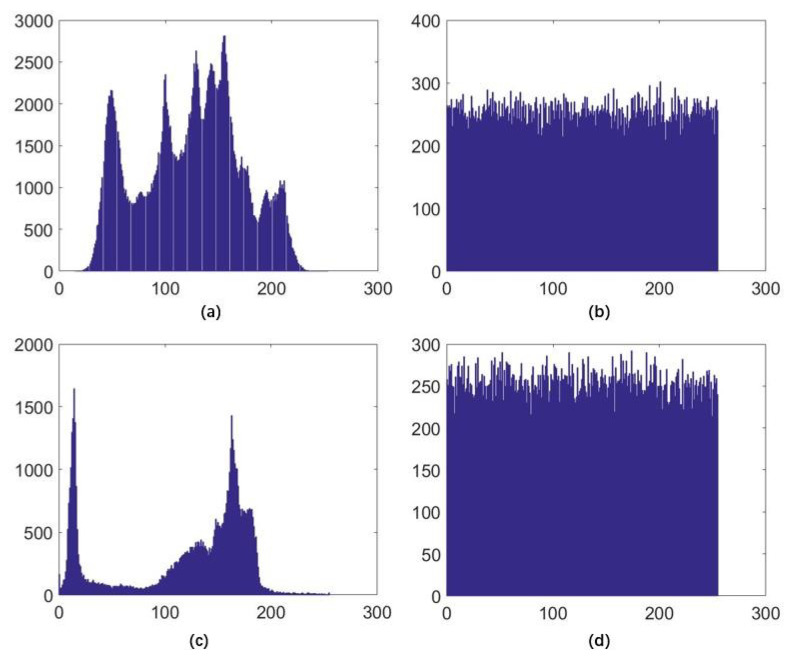
Histograms. (**a**,**b**) are histograms of the Lena image and the encrypted Lena image. (**c**,**d**) are histograms of the Cameraman image and the encrypted Cameraman image.

The histogram of digital image shows the distribution information of the pixel values. The histogram of an effective encrypted image should be significantly different from that of a plaintext image, which indicates that the ideal histogram of the encrypted image is evenly distributed, and the attacker cannot obtain any statistical information from the encrypted image. Figure 12 shows the histogram of the standard Lena and Cameraman images. The direct chart features of the original image are clear, while the histogram of the encrypted image is evenly distributed, and the attacker will not be able to obtain any useful information from the histogram.

#### 4.2.2. Correlation Analysis

Equation (5) is a method for calculating the correlation coefficients between adjacent pixels.

(5) $$p_{xy}\;=\;\frac{E\left\{\left[\left(x-E\left(x\right)\right]\left[y-E\left(y\right)\right]\right\}\right.}{D\left(x\right)\;\;D\left(y\right)}$$

where *E(x)* is the average value of pixels and *D(x)* is the variance of the pixels.

Generally speaking, the correlation between adjacent pixels in plaintext images is very strong; thus, a good encryption scheme should minimize the correlation between adjacent pixels. We randomly selected 2000 adjacent pixels in three directions: horizontal, vertical and diagonal. The correlation coefficients of the plaintext image and its encrypted image in three directions were obtained using Formula (5). Table 1 shows the correlation coefficients before and after the encryption of different images. Table 2 shows the correlation coefficients between the encryption scheme proposed in this section and the schemes proposed in other studies. Compared with references [19,34,35], it can be seen that the algorithm of dynamic correlation with plaintext pixels proposed in this paper encrypts the image, and the correlation coefficient of the image is greatly reduced. The correlation coefficients in the vertical and diagonal directions are smaller than those in the references. This means that the encryption effect is better.

**Figure 13:**
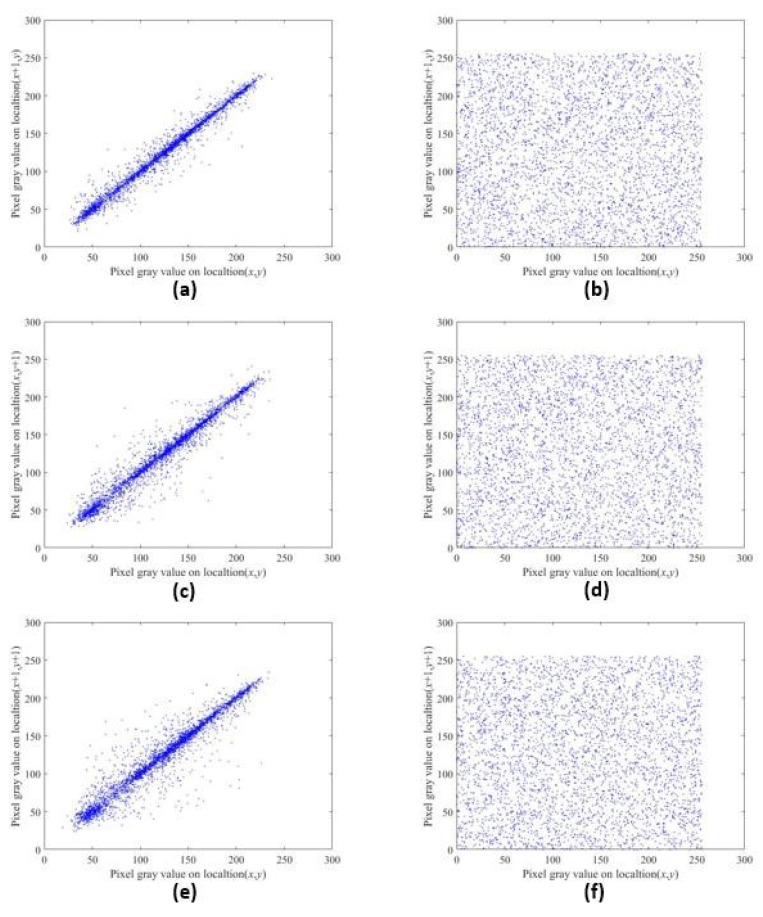
Distributions of the adjacent pixels in the original images and encrypted images of Lena. (**a**,**c**,**e**) are the distributions of the original image in the horizontal, vertical and diagonal directions, respectively. (**b**,**d**,**f**) are the distributions of the encrypted images in the horizontal, vertical and diagonal directions, respectively.

Figure 13 shows the distribution of the adjacent pixels of the original plaintext image and the encrypted image. We randomly selected 4000 pairs of horizontal, vertical and diagonal adjacent pixels, and drew their pixel distributions. It can be clearly seen that after using our encryption algorithm, the correlation of the ciphertext image has been significantly reduced, and the pixel values are diffused in the entire pixel value interval. No statistical information can be obtained through pixel distribution, which means that the algorithm in this paper has a good ability to resist statistical attacks.

### 4.3. Key Space Analysis

Generally speaking, the larger the key space, the higher the security of the algorithm. In this section, FL-NCHM as a hyperchaotic system, its initial parameters and initial values μ, λ, x${}_{0}$ and y${}_{0}$. they are generated by using the hash algorithm to perform operations on the plain text image, the operation results and values *μ*${}_{0}^{{}^{\prime }}$, *λ*${}_{0}^{{}^{\prime }}$, *x*${}_{0}^{{}^{\prime }}$ and *y*${}_{0}^{{}^{\prime }}$ performed a linear combination. The hash value obtained by the hash algorithm is a 256-bit binary number. Therefore, the key space size is 2${}^{256}$. In addition, if *x*${}_{0}^{{}^{\prime }}$ and *y*${}_{0}^{{}^{\prime }}$ are transmitted as part of the key, the key space will be greater than 2${}^{256}$. Considering the computing power of current computers, the key space of our proposed encryption algorithm is effective enough to prevent brute force attacks.

### 4.4. Sensitivity Analysis

#### 4.4.1. Key Sensitivity

For the image-encryption algorithm, key sensitivity is an important characteristic index, which can ensure the security of the algorithm against brute force attacks. In this section, due to the dynamic correlation between the key and the plaintext pixels, the tiny change of the plaintext image will produce two completely different encrypted images. As LF-NCHM is a hyperchaotic system that is very sensitive to the initial value and initial parameters, the slight change of the key will make the hyperchaotic sequence completely different, and thus two completely different encrypted images will be generated, and the encrypted image cannot be decrypted correctly.

**Figure 14:**
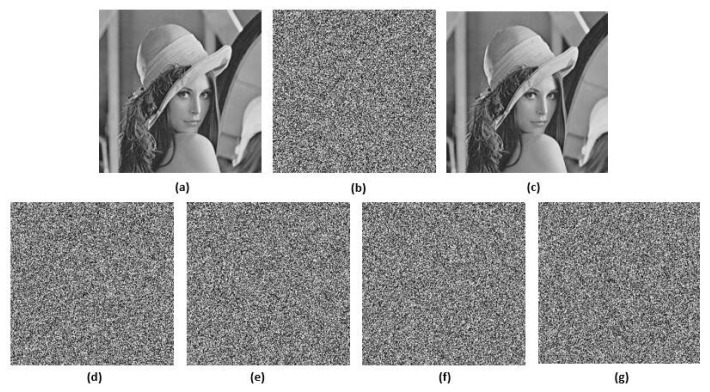
The key sensitivity test results. (**a**) Lena, (**b**) the encrypted image of Lena, (**c**) the decrypted image of Lena, (**d**) the parameter μ is modified, (**e**) the parameter λ is modified, (**f**) the parameter x${}_{0}$ is modified and (**g**) the parameter y${}_{0}$ is modified.

Figure 14 shows the plaintext image of Lena, the result of correctly encrypting and decrypting the image, and decrypting after changing one parameter of the decryption key 10${}^{-15}$. It can be seen from the image that no matter how small a parameter change is, no effective image information can be decrypted. Therefore, the algorithm proposed in this paper has strong key sensitivity.

#### 4.4.2. Differential Attack

There are qualitative and quantitative methods to measure the difference between two images of the same size. In this paper, we performed quantitative analysis. A good encryption algorithm should be sensitive to the subtle changes of a plaintext image. Even if only one bit of the plaintext image is changed, a completely different encrypted image should be obtained. Therefore, we first encrypted the plaintext image to obtain the encrypted image C. Second, an arbitrary pixel in the plaintext image was changed. Third, the changed plaintext image was encrypted to obtain the encrypted image C’. Finally, the number of pixels change rate (NPCR), the uniform average changing intensity (UACI) and the block average changing intensity (BACI) were calculated and compared with the theoretical values to see the difference between the two images. Table 3 shows the results of different images after the above operations. Their values are very close to the theoretical values, which indicates that our algorithm can well resist differential attacks. The three indicators are calculated as follows (6)–(8).

(6) $$NPCR\left(P_{1},P_{2}\right)=\frac{1}{MN}\sum_{i=1}^{M}\sum_{j=1}^{N}\left|Sign\left(P_{1}\left(i,j\right)-P_{2}\left(i,j\right)\right)\right|\times 100\%$$

(7) $$UACI\left(P_{1},P_{2}\right)=\;\frac{1}{MN}\sum_{i=1}^{M}\sum_{j=1}^{N}\frac{\left|P_{1}\left(i,j\right)-\;P_{2}\left(i,j\right)\right|}{255-0}\;\;\times 100\%$$

(8) $$BACI\left(P_{1},P_{2}\right)=\;\frac{1}{\left(M-1\right)\left(N-1\right)}\sum_{i=1}^{\left(M-1)(N-1\right)}\frac{m_{i}}{255}\;\;\times 100\%$$

where M*N is the image size. Sign(x) is the symbol function. m${}_{i}$ is the average of the absolute values of the difference between the two elements.

Table 4 shows the comparison results of the pixel number change rate (NPCR) and uniform average change intensity (UACI) using the encryption algorithm proposed in this paper and the algorithms proposed in other literature. It can also be seen from the comparison results that compared with references [34,37,38], the NPCR index of the algorithm proposed in this paper is closer to the theoretical value and is optimal. On the UACI indicator, the algorithm is superior to the literature [34,38], and lower than the literature [37]. Combining these two indicators, the algorithm is more convincing in resisting differential attacks.

### 4.5. Information Entropy Analysis

Information entropy reflects the uncertainty of image information, which can be used as an important feature to evaluate encryption system. The greater the entropy, the greater the amount of information, i.e., the greater the uncertainty, the less visual information can be obtained from the image. The calculation of information entropy is shown in Equation (9).

(9) $$\;H\left(m\right)\;=\;-\sum_{i=0}^{L}p\left(m_{i}\right){log}_{2}p\left(m_{i}\right)$$

where *m* represents the source of the information. *L* is the number of grayscale levels of the image. The theoretical value of information entropy *H* is 8.

Table 5 shows the information entropy of the plaintext Lena, Cameraman, and 4.1.01 images and their corresponding encrypted images. Table 5 shows that the information entropy of each ciphertext image is very close to the theoretical value, while the information entropy of each plaintext image is quite different from the theoretical value. This means that our encryption algorithm seriously disturbs the pixel values of the image, and the encryption effect is very prominent.

### 4.6. Chosen-Plaintext Analysis and Known-Plaintext Analysis

In the scheme of scrambling and pixel dynamic correlation encryption proposed in this paper, because a hash function was used to calculate the value of plaintext image, when a certain bit of a pixel of plaintext image changes, a completely different dynamic key will be obtained. According to the generation rules of dynamic keys and initial values, the hyperchaotic sequence generated by LF-NCHM is directly affected—that is, the hyperchaotic sequence used in scrambling and the diffusion stage key stream. In the process of scrambling, the pixel position of the plaintext image is related to the pixel value. If a certain bit of the pixel value changes, the corresponding position of the corresponding pixel value will also change. Due to the ingenious location selection mechanism, all the pixel positions will change with it, so the scrambling strategy has a certain diffusion effect on the premise of ensuring the scrambling. This strategy will lead to the attacker being unable to obtain the key stream of decrypting other images from any encrypted image, and thus cannot decrypt the plaintext image. Therefore, the encryption scheme proposed in this paper can resist the attacks of selective plaintext and known-plaintext.

### 4.7. Encryption Efficiency

A good encryption scheme should not only have excellent security performance but also have high efficiency. That is to say, both the encryption effect and encryption efficiency should be considered. Based on our encryption scheme, we used the encryption time, encryption throughput (ET) and the number of cycles to measure the encryption efficiency of our algorithm. The calculation of the ET and the number of cycles is shown in Equations (10) and (11):

(10) $$ET=\;\frac{{image}_{size}\left(byte\right)}{{encryption}_{time}\left(second\right)}$$

(11) $$Number\;of\;cycles\;per\;byte=\;\frac{{CPU}_{speed}\left(Hertz\right)}{ET\left(byte\right)}.$$

Less time, a large encryption throughput and a small number of cycles represent a high encryption efficiency. The experimental environment of this paper was MATLAB R2016a, Inter (R) Core (TM) i7-7700HQCPU @ 2.80 GHz with 8 GB RAM on Windows 10. To test the above three indicators, we selected the Lena image with the size of 256 × 256 to encrypt 100 times to calculate the average values.

Table 6 shows the encryption time of our algorithm and other algorithms from previous studies. To compare the encryption efficiency, we calculated the ET and the number of cycles in these studies. Table 7 shows the comparison between the ET and the number of cycles. It can be seen from the results that the encryption scheme we proposed takes less time and consumes fewer machine cycles. Our algorithm encryption efficiency and literature [34,39] have improved. Compared with literature [40], we have sacrificed a certain amount of time and machine cycles in exchange for the improvement of encryption security. This shows that our algorithm is successful in terms of security and efficiency.

## 5. Conclusions

In this paper, we proposed a LF-NCHM hyperchaotic system based on the general logistic map and Feigenbaum map. Through the analysis of the Lyapunov index and permutation entropy, LF-NCHM was found to have a wide hyperchaotic range and rich dynamic behavior. Based on LF-NCHM, a novel image-encryption algorithm based on the dynamic correlation between scrambling and plaintext pixels was proposed. The algorithm consists of two stages: scrambling and diffusion. In the scrambling stage, the current pixel of the new sequence is related to the previous pixel value, and the slight change of the pixel value leads to a complete difference of the subsequent pixels of the sequence. Therefore, the method not only has the scrambling effect but also has a certain diffusion effect, thus enhancing the ability to resist the attack of selective text.

To improve the use rate of the chaotic sequence, the confusion sequence in the diffusion stage was the same chaotic sequence generated in the scrambling stage. It can make the correlation between pixels more complex that the current pixels were generally related to the pixels in the previous two directions. In the two-dimensional pixel plane of the image, we started from the top left corner, left to right, top to bottom, so that the current pixel was related to its left side and upper side; then the second round was carried out, starting from the lower right corner, right to left, bottom to top diffusion, so that the current pixel was related to its right side and lower side.

The results showed that the algorithm could effectively resist statistical analysis attacks, differential attacks and plaintext selection attacks. Through the analysis of encryption speed, throughput and the number of machine cycles consumed, the time and space complexity of the algorithm is acceptable.

## Figures and Tables

**Figure 9:**
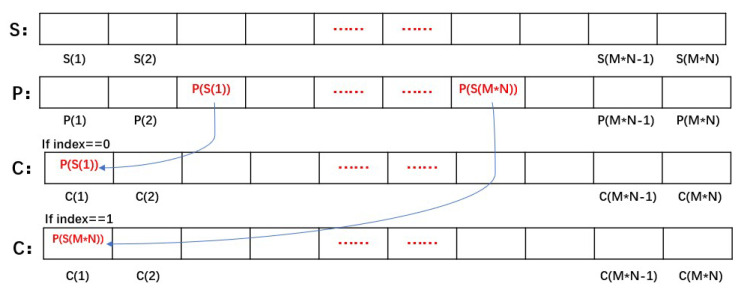
Example of Step 6.

**Table 1:** Correlation coefficients of different images.

	Horizontal	Vertical	Diagonal
Lena	0.9874	0.9973	0.9662
Encrypted Lena	0.0009	−0.0006	0.0007
Cameraman	0.9606	0.9355	0.9001
Encrypted Cameraman	0.0181	0.0004	−0.0024

**Table 2:** Correlation coefficients of the encrypted Lena images from different algorithms.

	Horizontal	Vertical	Diagonal
Lena	0.9874	0.9973	0.9662
Encrypted Lena	0.0009	−0.0006	0.0007
[34]	0.0008	0.0015	0.0032
[19]	0.0024	−0.0086	0.0402
[35]	0.0030	−0.0024	−0.0013

**Table 3:** The plaintext sensitivity analysis results.

Index	Lena	Cameraman	4.1.01	Theoretical Value
NPCR	99.6078	99.5575	99.6307	99.6094
UACI	33.4404	33.4058	33.4920	33.4635
BACI	26.7386	26.8189	26.6824	26.7712

**Table 4:** The plaintext sensitivity analysis results for different algorithms.

	NPCR	UACI
Ours	99.6078	33.4404
[34]	99.6262	33.4384
[37]	99.8214	33.4636
[38]	99.5727	33.4838

**Table 5:** The information entropy of different image information.

	Information Entropy
Lena	7.4486
Encrypted Lena	7.9970
Cameraman	7.1048
Encrypted Cameraman	7.9974
4.1.01	6.8981
Encrypted 4.1.01	7.9973

**Table 6:** The encryption time for different algorithms (seconds).

	256 × 256	512 × 512	Platform
Our	0.2679	1.1985	Matlab
[34]	0.2695	1.1869	Matlab
[39]	0.4389	1.8112	Matlab
[40]	0.039	0.156	Matlab

**Table 7:** A comparison of the efficiency of different algorithms.

	Encryption Throughput (MBps)	Cycles per Byte
Our	0.2333	12,001.71
[34]	0.2319	10,692.34
[39]	0.1424	23,440.03
[40]	1.6025	1368.76
